# Antioxidant Effects of *Baoyuan* Decoction on Dysfunctional Erythrocytes in High-Fat Diet-Induced Hyperlipidemic ApoE^−/−^ Mice

**DOI:** 10.1155/2019/5172480

**Published:** 2019-03-18

**Authors:** Zhen Wu, Fengyu Jin, Lingxiao Wang, Yunfang Zhao, Yong Jiang, Jun Li, Pengfei Tu, Jiao Zheng

**Affiliations:** ^1^Modern Research Center for Traditional Chinese Medicine, School of Chinese Materia Medica, Beijing University of Chinese Medicine, Beijing 100029, China; ^2^State Key Laboratory of Natural and Biomimetic Drugs, School of Pharmaceutical Sciences, Peking University, Beijing 100191, China

## Abstract

*Baoyuan* decoction (BYD), a traditional representative formula, has a long usage history in the treatment of cardiovascular diseases. Since the hyperlipidemia-induced dysfunction of erythrocyte is one of the most important causes of cardiovascular diseases, the improving effects of BYD against high-fat diet (HFD) induced the physiological and physical function of the erythrocytic injury and the potential mechanisms were deeply researched in this study. After 6 weeks of drug treatment, all doses of BYD had significantly decreased the lipid peroxidation in plasma of HFD-induced ApoE^−/−^ mice, even if it had not improved the lipid levels. Then, the erythrocyte-related experimental results showed that BYD had reduced erythrocyte osmotic fragility, stabilized erythrocyte membrane skeleton protein 4.2, and reformed the erythrocyte morphological changes by decreasing erythrocyte membrane lipid peroxidation levels. This study demonstrated that BYD may ameliorate the physiological and physical function of erythrocyte in hyperlipidemic mice through the antioxidant effect on erythrocyte membranes.

## 1. Introduction

Hyperlipidemia as a kind of abnormal lipid metabolism in the blood is one of the most common and important risk factors in the development of atherosclerosis and the resulting cardiovascular diseases [1]. Dysfunction of erythrocyte has been reported in hyperlipidemic patients and animal models [2, 3]. The lipid composition and the network of membrane-associated proteins together regulate the characteristic shape and elastic properties of the erythrocyte. The elevated level of cholesterol in plasma can enter into the erythrocyte membranes through lipoprotein exchange [4]. Moreover, the polyunsaturated fatty acids (PUFA) on erythrocyte membranes are also easily attacked by free radicals and the formation of lipid peroxide results in the changes of the membrane composition [5]. Furthermore, hyperlipidemia may affect the morphological structure and function of erythrocytes, such as erythrocyte morphology, osmotic fragility, skeletal protein, lipid content of erythrocyte membrane, and lipid peroxidation. Therefore, the damage of erythrocyte by oxidative stress induced by hyperlipidemia is one of the important pathophysiological bases of cardiovascular diseases.

*Baoyuan* decoction (BYD) is a traditional representative formula, which is composed of *Panax ginseng* C. A. Mey. (Chinese name: *Renshen*), *Astragalus membranaceus* (Fisch.) Bunge var. *mongholicus* (Bunge) Hsiao (*Huangqi*), *Glycyrrhiza uralensis* Fisch (*Zhigancao*), and *Cinnamomum cassia* Presl (*Rougui*). Our previous researches proved that the clarification of the chemical composition of BYD was very complex [6–8]. Up to now, 236 compounds were identified, including saponin compounds, procyanidins, flavonoids, terpenoids, and lignans. Modern pharmacological studies have demonstrated that BYD has the curative effect and high safety in the treatment of heart failure, coronary heart disease, and other cardiovascular diseases [9, 10]. However, its effect on the hyperlipidemia-induced dysfunction of the erythrocyte is not thoroughly investigated. In the present study, we sought to examine the improvement of BYD on the function of erythrocyte in hyperlipidemic ApoE^−/−^ mice and to provide novel information for the clinical application and future studies on the mechanisms of BYD.

## 2. Materials and Methods

### 2.1. Experimental Animals and Drug Treatments

Male ApoE^−/−^ mice on a C57BL/6J background (SPF grade), weighing 18~22 g, were purchased from Beijing Vital River Laboratory Animal Technology Co. Ltd. (license no. SCXK (Jing), 2016-0011). All mice were raised in the barrier environment (room temperature of 24 ± 1°C and relative humidity of 50 ± 1% with a 12 h day/night cycle) and adaptability fed 1 week. Then animals were randomly divided into 5 groups (*n* = 6~9): chow diet control group (CG), HFD-induced model group (HG), the low and high doses of BYD-treated group (BYD-L and BYD-H, respectively), and ezetimibe (Schering-Plough Ltd., USA, lot: 2EZPA17005)-treated group (EG). According to the daily dose of the adult, mice assigned to BYD-L, BYD-H, and EG groups were orally administrated 150 and 300 mg/kg/day of BYD and 10 mg/kg/day of ezetimibe. In addition, in the control group, mice were fed with chow diet; the other groups of mice were fed high-fat diet (HFD) to establish the model of hyperlipidemic mice. The HFD contained 89.8% chow diet, 0.2% cholesterol, and 10% fat. BYD was administered orally by gavage for 6 weeks while maintaining HFD. All animal experiments have followed the guidelines of the ethics committee of Beijing University of Chinese Medicine. And the experimental protocol was approved by the medical animal experiment ethics committee of Beijing University of Chinese Medicine.

### 2.2. Medicinal Materials

Four crude materials of BYD were purchased from Anguo TCM market (Hebei, China) and were authenticated by Prof. Pengfei Tu. It was prepared by combining astragalus roots, ginseng, liquorice, and cinnamon with a ratio of 6 : 2 : 2 : 1. The extraction method and chemical profile were performed according to the previous researches [6]. Lyophilized BYD powder was entirely dissolved in ultrapure water (18.2 MΩ), from a Milli-Q water purification system (Millipore, France), for animal experiments.

### 2.3. Plasma Lipid Profiles and Malondialdehyde (MDA) Analysis

All mice underwent fasting for 6 hours before collecting blood samples. The blood samples were collected from the retro-orbital sinus and anticoagulated by heparin, centrifuged at 3000 g for 10 minutes at 4°C. According to the instructions of the kit (Applygen Technologies Inc., Beijing, China) manufacturers, plasma levels of total cholesterol (TC) and triglyceride (TG) were measured using the GPO-PAP method. The polyethylene glycol (PEG) precipitation method was used for determining high-density lipoprotein cholesterol (HDLC). The plasma MDA levels were determined by the commercial assay kit (Nanjing Jiancheng Bioengineering Institute, Nanjing, China).

### 2.4. Measurement of Erythrocyte Osmotic Fragility

2 *μ*L of anticoagulant whole blood was mixed with 200 *μ*L of each dilution of phosphate buffered solution (PBS) and incubated for 1 hour at room temperature. After blood samples were centrifuged at 3000 g for 6 minutes, the optical density of supernatant was determined at 540 nm. The complete hemolytic erythrocyte sample, in distilled water, was used as the positive control, and the nonhemolytic erythrocyte samples under PBS of normal osmotic pressure were used as the negative control [11]. The hemolytic rate of erythrocyte at different osmotic pressure was calculated.

### 2.5. SDS-PAGE (Sodium Dodecyl Sulfate-Polyacrylamide Gel Electrophoresis) and MDA Analysis of Erythrocyte Membranes

Whole blood samples were centrifuged at 3000 g for 10 minutes at 4°C to collect the lower erythrocyte, discard plasma, and buffy coats [12]. Each sample was washed 3 times with PBS of normal osmotic pressure, then a certain amount of erythrocyte samples was added to 20× volume hypotonic PBS and placed in −80°C for 15 minutes, repeated freezing and thawing to make the erythrocyte rupture completely. Collected erythrocyte membranes, centrifuged at 12000 g for 10 minutes at 4°C, were washed 3 times with hypotonic PBS until the ghost became white. Erythrocyte membrane samples were dissolved in distilled water and the protein concentrations were quantified according to the method of BCA by the commercial assay kit (Applygen Technologies Inc., Beijing, China). Erythrocyte membranes were added to a 3× volume sample buffer solution and mixed evenly. The protein was denatured by heating at 95°C for 10 minutes and separated by NuPAGE™ 4~12% Bis-Tris Gel (Life Technology™, CA, USA). Protein marker was used to label the molecular weight. Then the gel was dyed with Coomassie brilliant blue staining [13]. The MDA levels of erythrocyte membranes in each group were determined using the TBA method by the commercial assay kit (Nanjing Jiancheng Bioengineering Institute, Nanjing, China).

### 2.6. Extraction and Measurement of Erythrocyte Ghost Lipids

Erythrocyte membrane lipids were extracted according to the previous studies [14]. A certain amount of erythrocyte membrane solution was added into a methanol-dichloromethane solvent system. After 1 minute of vortex, the solution was centrifuged at 800 g for 10 minutes. Then the lower layer of dichloromethane was separated into the new tube. In order to prevent the auto-oxidation of lipids in the extraction process, all solvents were added butylated hydroxytoluene (BHT, 0.001%, w/v) as the antioxidant [15]. Lipids were dissolved with 5% Triton X-100 after being dried with nitrogen. The levels of TC, free cholesterol (FC), cholesterol ester (CE), and phospholipids (PL) in extracted lipids were measured by the commercial assay kit (Applygen Technologies Inc., Beijing, China).

### 2.7. Western Blot Analysis of Erythrocyte Ghost Protein

The quantified membrane protein was separated by SDS-PAGE and transferred to the polyvinylidene fluoride membrane (Merck Millipore, Billerica, MA, USA). After being blocked with 5% skim milk for 1.5 hours at room temperature, blots were incubated overnight at 4°C with the following primary antibodies: anti-4-HNE (4-hydroxynonenal) (Abcam, ab46545); anti-SOD2 (superoxide dismutase2) (Santa, sc-137254); anti-SOD3 (superoxide dismutase3) (Santa, sc-271170); anti-4.1R (Santa, sc-166759l); anti-p55 CDC (Santa, sc-13162); and anti-Na, K-ATPase (CST, #3010). After that, the membranes were incubated with HRP-conjugated secondary antibody at 4°C for 4 hours. The protein bands were visualized using ECL reagents (Applygen Technologies Inc., Beijing, China) and were quantified with ImageJ software.

### 2.8. Erythrocyte Morphology Analysis

Fresh erythrocytes were fixed in the 2.5% glutaraldehyde solution at 4°C for 2 hours, rinsed with 0.1 M PBS 3 times. Then samples were dehydrated through graded ethanol (50%~100%, v/v), replaced with isoamyl acetate, and dripped onto the clean glass slide. After drying, carbon gold was sprayed to observe erythrocyte morphology under scanning electron microscopy (SEM) [16].

### 2.9. Statistical Analysis

The data were presented as means ± SEM. One-way ANOVA was used to compare the significant differences for multiple comparisons, followed by Student's *t*-test for two groups as appropriate. All statistical analyses were performed with Graphpad Prism 6.02 (GraphPad, San Diego, CA). *P* < 0.05 was considered statistically significant.

## 3. Results

### 3.1. Changes in Plasma Lipid Profiles and MDA in the HFD-Induced Mice

**Figure 1:**
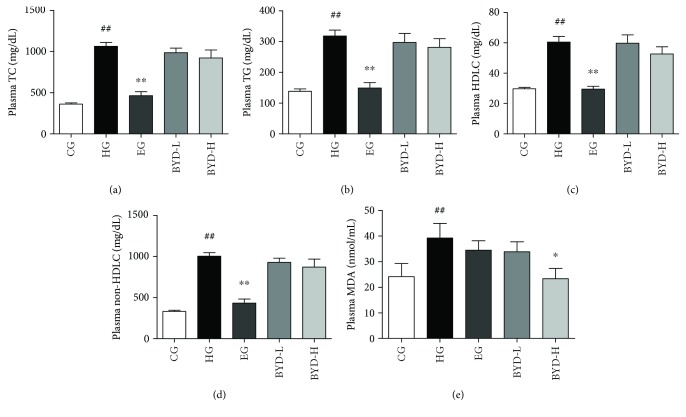
Plasma lipid profiles and MDA levels after 6 weeks of BYD treatment in ApoE^−/−^ mice (*n* = 6—10). Levels of TC (a), TG (b), HDLC (c), non-HDLC (d), and MDA (e) in mice plasma. ^##^ *P* < 0.01 compared with CG; ^∗^ *P* < 0.05 and ^∗∗^ *P* < 0.01 compared with HG. TC: total cholesterol; TG: triglyceride; HDLC: high-density lipoprotein cholesterol; MDA: malondialdehyde; BYD: *Baoyuan* decoction; CG: chow diet control group; HFD: high-fat diet; HG: HFD-induced model group; EG: ezetimibe-treated group; BYD-L: low dose of BYD-treated group; BYD-H: high dose of BYD-treated group.

The C57BL/6J ApoE^−/−^ mice on HFD were treated with vehicle (0.5% CMCNa), ezetimibe (10 mg/kg/day), or BYD (150 or 300 mg/kg/day) for 6 weeks. Ezetimibe is an inhibitor of cholesterol absorption with cholesterol-lowering effect [17], BYD was compared with it. Before drug treatment, animals were fed HFD for one week, which significantly increased TC (*P* < 0.01) and non-HDLC (*P* < 0.01) levels almost 3 times (data not shown). After 6 weeks of drug treatment, no obvious toxicity of all doses of BYD and ezetimibe was observed. As for the lipid levels, the treatment of these hyperlipidemic mice with ezetimibe by oral administration resulted in a remarkable decrease in plasma TC, TG, HDLC, and non-HDLC levels (*P* < 0.01, Figure 1). However, two doses of BYD treatment had not shown any effect on plasma lipid levels. To explore the antioxidation effect of BYD and ezetimibe, we further analyzed the MDA levels in plasma. MDA is a natural lipid peroxidation product, produced by the action of oxygen free radicals on lipid *in vivo* [18]. Interestingly, BYD then at 300 mg/kg/day dose treatment significantly decreased the MDA levels by 40.42% (*P* < 0.05, Figure 1(e)) compared with the HG mice. Then we examined the antioxidation activity of BYD by inhibiting LDL oxidation and free radical scavenging effect *in vitro*. As shown in Figures S1, S2, and S3, BYD significantly improved the CuSO_4_-induced LDL oxidation and promoted the free radical scavenging activity in a dose-dependent manner. All the data showed that BYD had the significant antioxidant capacity *in vivo* and *in vitro*.

### 3.2. Effect of BYD on Erythrocyte Osmotic Fragility

**Figure 2:**
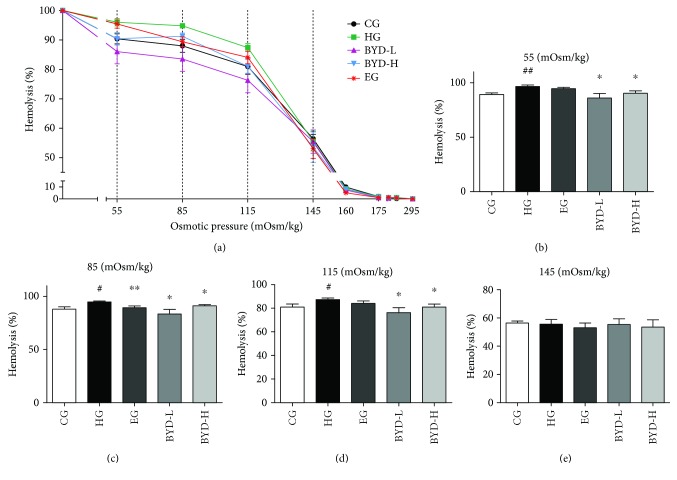
BYD improved the osmotic fragility of erythrocyte in HFD-induced mice (*n* = 4—7). (a) Osmotic fragility curves of all drug-treated groups. The percentage of hemolysis of erythrocyte under 55 mOsm/kg (b), 85 mOsm/kg (c), 115 mOsm/kg (d), and 145 mOsm/kg (e). ^#^ *P* < 0.05 and ^##^ *P* < 0.01 compared with CG; ^∗^ *P* < 0.05 and ^∗∗^ *P* < 0.01 compared with HG.

Since dysfunction of the erythrocyte is considered to be an important factor in the development of cardiovascular diseases [19], we studied the effect of BYD on erythrocyte functions by osmotic fragility test. Hyperlipidemic erythrocytes with lipid peroxidation are osmotically fragile cells that rupture more easily in a hypotonic solution than normal erythrocytes [15, 20]. In our research, the data showed that all the erythrocytes remained intact at 295 mOsm/kg PBS solution in all groups, as shown in Figure 2. The percentage of fragmentation rose with the decrease of the osmotic press. From 115 to 55 mOsm/kg osmotic press, more erythrocytes appeared fragmentized in HG mice than in CG mice. The erythrocytes of all BYD-treated mice had the left-shifted osmotic fragility curve, indicating that the BYD treatments significantly improved the osmotic fragility of erythrocytes compared to those of HG mice (Figure 2(a)). Moreover, at the osmotic pressure of 55, 85, and 115 mOsm/kg, all doses of the BYD treatment had remarkable antihemolysis effects on hyperlipidemic mice compared with the HG mice (*P* < 0.05, Figures 2(b)–2(d)). Besides, even at a high concentration (1 mg/mL), BYD did not affect the osmotic fragility in normal erythrocyte *in vitro* (Figure S3). These results suggested that BYD significantly restricted the increase in erythrocyte osmotic fragility which is induced by hyperlipidemia.

### 3.3. BYD Stabilized Erythrocyte Membrane Skeletal Proteins of HFD-Induced Mice

**Figure 3:**
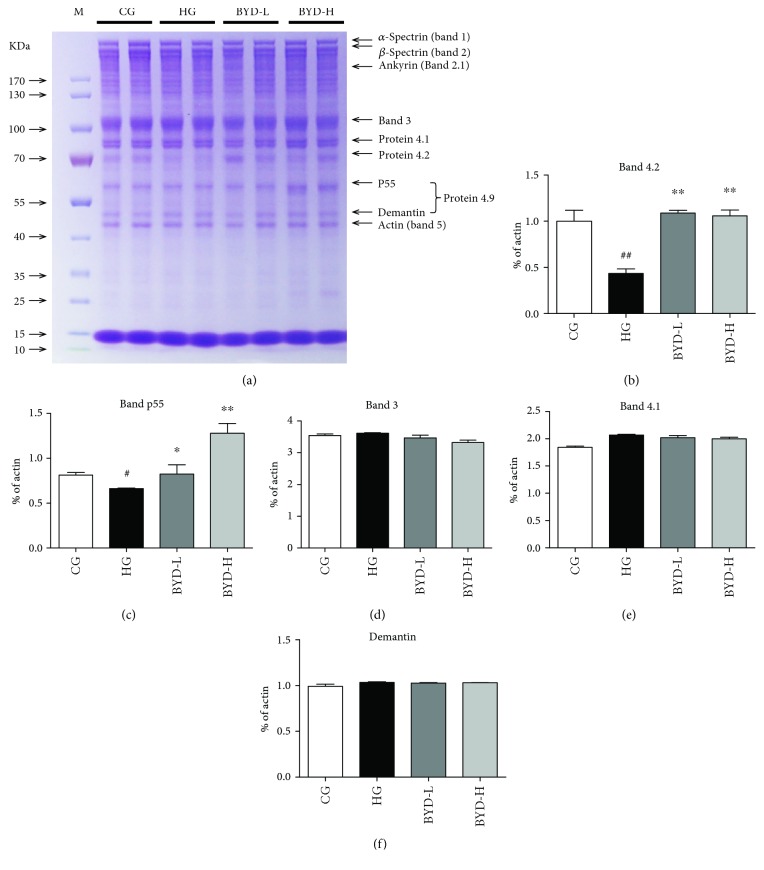
Protein expressions of erythrocyte membrane skeleton after BYD treatment (*n* = 4). (a) SDS-PAGE analysis of the erythrocyte skeletal protein in mice. The relative quantification of skeletal protein by SDS-PAGE, including band 3 (b), band 4.1 (c), band 4.2 (d), band p55 (e), and Demantin (f). Actin was used as internal control. “M” meant protein marker to indicate molecular weight. ^#^ *P* < 0.05 and ^##^ *P* < 0.01 compared with CG; ^∗^ *P* < 0.05 and ^∗∗^ *P* < 0.01 compared with HG.

Based on the previous researches, the osmotic fragility of erythrocyte could be reflected in the membrane ability to maintain structural integrity, such as abnormal shape, the degree of peroxide, membrane lipid, and protein compositions [21]. To investigate the mechanism of the improvement of BYD on erythrocyte membrane fragility, erythrocyte ghost's protein had been studied by SDS-PAGE firstly (Figure 3). Our analysis of erythrocyte indicated skeletal proteins of erythrocyte ghosts mainly referred to the spectrin, ankyrin, band 3, protein 4.1, protein 4.2, actin, etc. (Figure 3(a)). Then we found a significant reduction of 72/74 kDa proteins (protein 4.2) and a modest decrease of 55 kDa proteins (protein p55) in ghosts' fractions of HG mice compared with those of CG mice (Figure 3(a)). As for the drug treatment, BYD remarkably increased the levels of protein 4.2 and p55 in erythrocyte ghosts dose-dependently (Figures 3(b) and 3(c)). There were no significant alterations in the amounts of spectrin, ankyrin, band 3, protein 4.1, and actin either in HFD or BYD-treated erythrocytes. Together, our results suggested that BYD significantly improved levels of protein 4.2 and p55 in erythrocyte ghosts when they were subjected to HFD.

**Figure 4:**
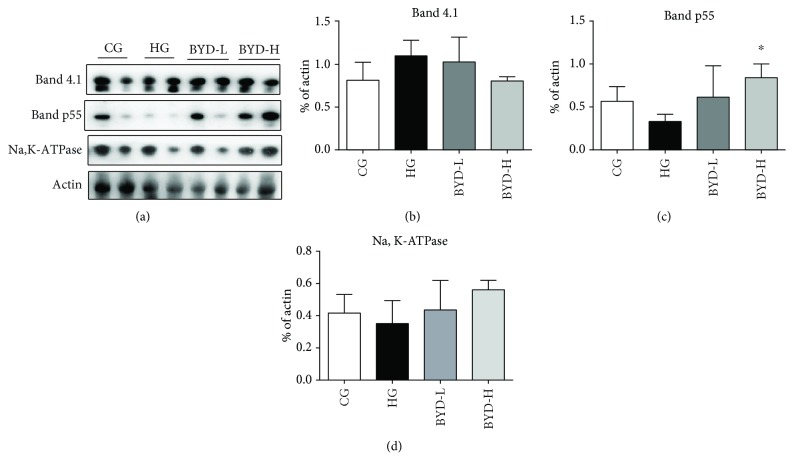
Western blotting analysis of band 4.1, P55, Na/K ATPase, and actin in erythrocyte membrane skeleton after BYD treatment (*n* = 4). (a) The protein bands of band 4.1, band p55, Na, K-ATPase, and actin were visualized using ECL reagents. The relative quantifications of band 4.1 (b), band p55 (c), and Na, K-ATPase (d). Actin was used as internal control. ^∗^ *P* < 0.05 compared with HG.

In order to confirm the results of erythrocyte ghosts' skeletal proteins in SDS-PAGE, we tested the protein expressions of band 4.1, p55, actin, and Na, K-ATPase in erythrocyte ghosts by western blot. There were no significant alterations in the amounts of band 4.1, actin, and Na, K-ATPase of erythrocyte ghosts in all mice (Figures 4(a), 4(b), and 4(d)). Consistent with the result of SDS-PAGE, the protein levels of band p55 in erythrocyte ghosts appeared to be remarkably reduced in the erythrocyte ghosts of HG mice, compared with those of CG mice. Also, band p55 protein values of the two doses of BYD-treated mice became higher after the therapy compared to those of HG mice (Figure 4(c)). In our western blot, we were unable to detect any band 4.2 in erythrocyte ghosts presumably because of the low reactivity of the polyclonal antibody directed against band 4.2.

### 3.4. BYD Inhibited the Lipid Peroxidation of Erythrocyte

**Figure 5:**
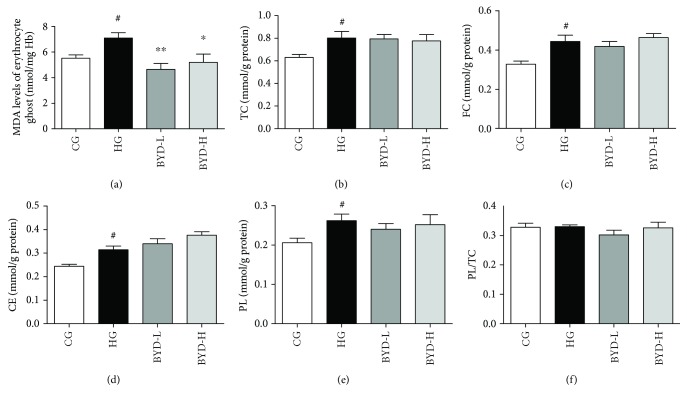
Lipid contents and lipid peroxidation of the erythrocyte ghosts after BYD treatments in ApoE^−/−^ mice (*n* = 5). (a) MDA levels in erythrocyte ghosts. Contents of TC (b), FC (c), CE (d), PL (e), and PL/TC (f) in erythrocyte ghosts. Results were normalized to membrane protein contents. ^#^ *P* < 0.05 compared with CG; ^∗^ *P* < 0.05 and ^∗∗^ *P* < 0.01 compared with HG. CE: cholesteryl ester.

Besides the erythrocyte membrane skeletal protein, the lipid and lipid peroxidation levels are also the important factors associated with the improved osmotic fragility in erythrocyte. Cholesterol enrichment in erythrocyte may cause impairment of osmotic fragility that may accelerate atherosclerotic lesions [21, 22]. As for lipid peroxidation, the crystallized cholesterol formed in the extracellular milieu of the plasma membrane contributes directly to the pathogenesis of cardiovascular diseases. Then the lipid and lipid peroxidation levels were studied by the following tests. In addition to the PL/TC ratio (Figure 5(f)), higher MDA (Figure 5(a)), TC (Figure 5(b)), FC (Figure 5(c)), CE (Figure 5(d)), and PL (Figure 5(e)) levels of erythrocyte ghosts in hyperlipidemic mice (HG) compared to those in chow diet mice (CG) were found after 6 weeks of HFD induction. However, only the MDA levels of erythrocyte ghosts were significantly reduced by BYD.

**Figure 6:**
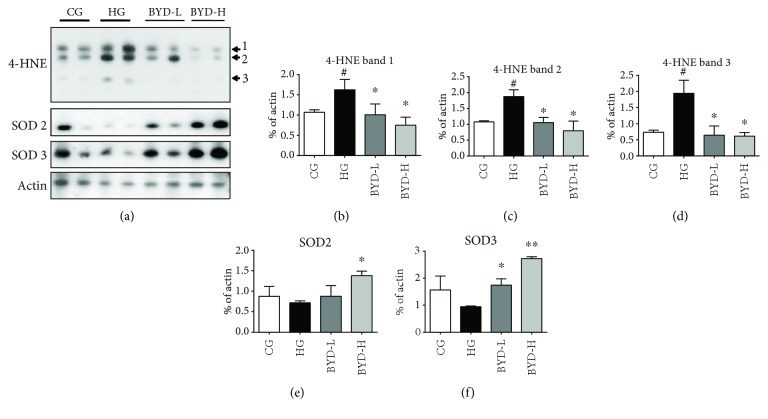
Western blot analysis of oxidation-related proteins in erythrocyte ghosts (*n* = 4). (a) The protein bands of 4-HNE, SOD2, and SOD3 were visualized using ECL reagents. Relative quantifications of 4-HNE band1 (b), 4-HNE band2 (c), 4-HNE band3 (d), SOD2 (e), and SOD3 (f). Results were normalized to actin. ^#^ *P* < 0.05 compared with CG; ^∗^ *P* < 0.05, ^∗∗^ *P* < 0.01 compared with HG. 4-HNE: 4-hydroxynonenal; SOD2: superoxide dismutase2; SOD3: superoxide dismutase3.

To gain insight into the mechanism of BYD on MDA-lowering effect in erythrocyte membrane, the effect and specificity of BYD on antilipid peroxidation processing were directly examined (Figure 6). Since a variety of harmful fragmentation products are formed during the oxidative process, including 4-HNE [23], the 4-HNE levels in erythrocyte ghosts of different groups were investigated firstly. The increased 4-HNE levels of erythrocyte ghosts, including bands 1, 2, and 3, had been found in HG mice compared with those in CG mice. Consistent with the decrease of MDA in erythrocyte ghost after BYD treatment, BYD remarkably inhibited the 4-HNE expression in a dose-dependent manner (*P* < 0.05, Figures 6(a)–6(d)). Because most of the superoxide is converted to hydrogen peroxide by SOD [24], then we investigated the SOD2 and SOD3 levels of erythrocyte ghosts. As shown in Figures 6(a), 6(e), and 6(f), the decreased SOD2 and SOD3 levels induced by hyperlipidemia were recovered by BYD dose-dependently; these results suggested that BYD improved erythrocyte osmotic fragility due to its antilipid peroxidation effect.

### 3.5. BYD Improved Erythrocyte Morphology of HFD-Induced Mice

**Figure 7:**
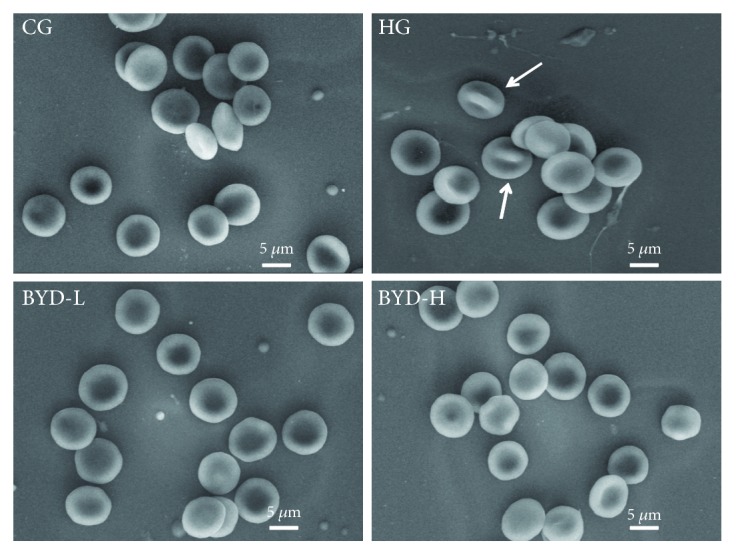
Erythrocyte morphology of ApoE^−/−^ mice after 6 weeks of BYD treatment was observed by SEM (*n* = 5). Scale bar, 5 *μ*M.

Normal erythrocyte morphology plays an important role in maintaining the body's homeostasis. Membrane lipids, proteins, and enzymes make up the visible components of the cell membranes. When the proportion of various parts of erythrocyte membranes is changed due to various factors, the morphology of erythrocyte must change as well. The erythrocyte morphology of HG mostly had the distinct characteristics of protrusion or irregular appearances (Figure 7, arrows), while the other groups showed typical double concave disk erythrocyte morphology. All data indicated that BYD improved erythrocyte morphology of hyperlipidemic mice, which could be related to the stabilization of erythrocyte membrane skeleton protein and the reduction of lipid peroxidation.

## 4. Discussion

It has been emphasized that the dysfunction of erythrocyte can play an important role in atherosclerosis and coronary heart disease [19]. Erythrocyte has a unique biological structure which contains high concentrations of PUFA [5, 25]. During their lifetime, erythrocytes travel through vessels for delivering oxygen to the tissues. For this reason, they might be expected to have high oxygen content [25, 26]. Then it is particularly vulnerable to be damaged due to oxidants. Although the erythrocyte has the efficient enzymatic machinery to convert hydrogen peroxide to water, the hyperlipidemia-induced high-level oxidant stress may exceed the erythrocyte reductive capacity. Since BYD has been used to treat cardiovascular diseases for a long period, this study was undertaken to determine whether BYD showed the improvement in hyperlipidemic erythrocyte dysfunction. Our results showed that although BYD had no effect on the lipid level, it dose-dependently decreased the MDA levels of plasma induced by hyperlipidemia. These results suggested that BYD could inhibit lipid peroxidation in spite of it having no lipid-lowering effect.

Osmotic fragility is one of the important physiological characteristics of erythrocyte, which mainly measures the tensile strength of erythrocyte in low-permeability environment [27]. The osmotic fragility is negatively correlated with the resistance of erythrocyte in the hypotonic solution. A great deal of studies showed that the osmotic fragility of erythrocyte was closely related to the fluidity of erythrocyte membranes and the properties of erythrocyte membrane skeleton, for instance, the insufficiency of protein 4.2 [28], the ratio of C : P [21], fatty acid composition of phospholipids [29], and lipid peroxidation [30]. The changes of membrane structure caused by various factors, especially the cholesterol-rich diet, can lead to the change of osmotic fragility. We found that osmotic fragility curves of erythrocyte in all doses in BYD-treated mice were shifted left in comparison to the HG; the underlying mechanism of it should be bound up with stabilizing the composition of erythrocyte membranes.

Erythrocyte membrane skeleton proteins play the significant role in maintaining the erythrocyte morphology, which is mainly composed of spectrin, ankyrin, band 3, protein 4.1, protein 4.2, actin, etc. The interaction of skeleton proteins, mosaic proteins, and membrane lipids collectively maintains the integrity of the membrane structure [31]. The SDS-PAGE result of erythrocyte ghosts showed that the levels of protein 4.2 and p55 were remarkably reduced after HFD feeding. Previous studies have indicated that the protein 4.2, as the anchoring protein, may play an important role in connecting the skeleton network of erythrocyte membranes to band 3 [32]. It probably has a role in erythrocyte shape and mechanical property regulation [33]. As for p55, subsequent studies established that the p55 protein is an obligate component of the protein 4.1-glycophorin C complex, which regulates the stability and mechanical properties of the erythrocyte membranes [34]. Then we suggested that the changes in erythrocyte osmotic fragility of hyperlipidemic mice should come from the decrease of band 4.2 and p55 in erythrocyte membranes. BYD significantly restored the reduction of band 4.2 and p55. Thus, our result indicated that BYD should improve the osmotic fragility and mechanical properties of the erythrocyte plasma membranes in hyperlipidemic mice by stabilizing erythrocyte membrane skeletal proteins.

Besides the erythrocyte membrane skeletal protein, previous studies have reported that the erythrocyte membrane contains approximately 40% lipids, including free cholesterol and phospholipids [35]. The relative amounts of cholesterol and phospholipids are responsible for the fluid properties of the erythrocyte membranes. Since it has lipid exchange between plasma and erythrocyte membranes, the lipid distribution in the erythrocyte had been found in hyperlipidemic patients and animal models [4, 36]. Consistent with the previous study [37], we also found that the increased plasma lipid levels upregulated the TC and FC levels in erythrocyte membranes. However, BYD didn't improve the lipid levels in erythrocyte membranes of hyperlipidemic mice.

Since erythrocyte membranes are rich in PUFA and hence are potential targets for oxidative modification, we researched the degree of lipid peroxidation in the following studies. Among the lipid peroxidation products, 4-HNE represents one of the most bioactive and well-studied lipid alkenals [38]. Then we studied erythrocyte membrane levels of 4-HNE, the products of lipid peroxidation by ROS, as well as SOD. In our studies, hyperlipidemia significantly induced the increase of 4-HNE level in erythrocyte membranes, and it was accompanied by a decrease of SOD2 and SOD3 proteins. As for the drug treatments, BYD reversed the increase of 4-HNE level and the decrease of SOD2 and SOD3 of ApoE^−/−^ mice erythrocyte membranes. These results suggested that BYD also efficiently lowered the lipid peroxidation in erythrocyte membranes by increasing the expressions of SOD2 and SOD3. When hyperlipidemia occurred, raised plasma lipoproteins such as LDL (low-density lipoprotein) and VLDL (very low-density lipoprotein) may stimulate leukocytes to produce ROS and then overload the antioxidant system [39]. The oxidative stress damages the erythrocyte membranes and impairs its osmotic fragility. Then the damaged erythrocytes deliver oxygen to the tissues to be transferred to cells, which come in contact with erythrocytes resulting in tissue damage [40]. SOD2 is an antioxidant protein that protects cells against mitochondrial superoxide. Because of its mitochondrial location, SOD2 is the principal defense against the toxicity of superoxide anions generated by the oxidative lipids [41]. There was no mitochondrion in mature erythrocytes; however, the expression of the SOD2 protein was detected by western blot, presumably due to the presence of undifferentiated erythrocytes. SOD3 is localized in the extracellular matrix of various tissues, especially blood vessel wall, but a smaller proportion is also found in plasma [42]. Our studies demonstrated that BYD inhibited the lipid peroxidation of erythrocyte membranes by activating the levels of SOD2 and SOD3, improving the cell osmotic fragility and function.

In the present study, the changes in erythrocyte morphology of hyperlipidemic mice were also worthy of our concern. Erythrocyte morphology is a crucial parameter in the field of rheology to maintain the body's homeostasis and vulnerability to many factors, including osmotic fragility, the composition of erythrocyte membrane skeleton protein, and lipid peroxidation induced by oxidative stress [43, 44]. We observed that the erythrocyte morphology of HG mostly had a distinct characteristic of the protrusion by the SEM, while the other groups showed typical double concave disk erythrocyte morphology. So the data suggested that BYD improved erythrocyte morphology of hyperlipidemic mice.

## 5. Conclusions

Since BYD has a curative effect and high safety in the treatment of heart failure, improvement of erythrocyte function was generally considered as one of the factors involved. Through the data accumulated from our research, we believed that hyperlipidemia may cause the abnormalities of erythrocyte in ApoE^−/−^ mice. Further study on BYD treatment had performed its improvement on erythrocyte osmotic fragility, membrane skeleton protein, and morphological changes by inhibiting lipid peroxidation of erythrocyte membranes. Therefore, we deemed that the potential mechanism of BYD in improving the physiological and physical function of erythrocyte is through its antioxidant effect.

## Data Availability

The data used to support the findings of this study are available from the corresponding author upon request.

## Abbreviations

4-HNE: 4-Hydroxynonenal
BHT: Butylated hydroxytoluene
BYD: Baoyuan decoction
BYD-H: High dose of BYD-treated group
BYD-L: Low dose of BYD-treated group
CE: Cholesteryl ester
CG: Chow diet control group
EG: Ezetimibe-treated group
FC: Free cholesterol
HDLC: High-density lipoprotein cholesterol
HFD: High-fat diet
HG: HFD-induced model group
LDL: Low-density lipoprotein
MDA: Malondialdehyde
PBS: Phosphate buffered solution
PEG: Polyethylene glycol
PUFA: Polyunsaturated fatty acids
SDS-PAGE: Sodium dodecyl sulfate-polyacrylamide gel electrophoresis
SEM: Scanning electron microscopy
SOD2: Superoxide dismutase2
SOD3: Superoxide dismutase3
TC: Total cholesterol
TG: Triglyceride
VLDL: Very low-density lipoprotein.

## Supplementary Materials

Supplementary Materials Figure S1: inhibition of CuSO_4_-induced LDL oxidation by BYD. Figure S2: scavenging effect on 2,2′-azino-bis (3-ethylbenzothiazoline-6-sulphonic acid) (ABTS+) radicals. Figure S3: scavenging effect on 1,1-diphenyl-2-picrylhydrazyl (DPPH) radicals. Figure S4: BYD was not toxic to normal erythrocytes in vitro.
