# Supplementary material for: Antioxidant Effects of Baoyuan Decoction on Dysfunctional Erythrocytes in High-Fat Diet-Induced Hyperlipidemic ApoE−/− Mice
Source: Oxid Med Cell Longev. 2019 Mar 18;2019:5172480. doi: 10.1155/2019/5172480 (PMC6476116; doi:10.1155/2019/5172480)
Supplement: Supplementary Materials — Figure S1: inhibition of CuSO4-induced LDL oxidation by BYD. Figure S2: scavenging effect on 2,2′-azino-bis (3-ethylbenzothiazoline-6-sulphonic acid) (ABTS+) radicals. Figure S3: scavenging effect on 1,1-diphenyl-2-picrylhydrazyl (DPPH) radicals. Figure S4: BYD was not toxic to normal erythrocytes in vitro. [file 5172480.f1.docx]

**Supplemental Information**

**Antioxidant Effects of *Baoyuan* Decoction on Dysfunctional Erythrocytes in High Fat Diet-Induced Hyperlipidemic ApoE^-/-^ mice**

**Zhen Wu^1^, Fengyu Jin^1^, Lingxiao Wang^1^, Yunfang Zhao^1^, Yong Jiang^2^, Jun Li^1^, Pengfei Tu*^1, 2^, Jiao Zheng*^1^**

*^1^ Modern Research Center for Traditional Chinese Medicine, School of Chinese Materia Medica, Beijing University of Chinese Medicine, Beijing 100029, People's Republic of China*

*^2^ State Key Laboratory of Natural and Biomimetic Drugs, School of Pharmaceutical Sciences, Peking University, Beijing 100191, People's Republic of China*

***Correspondence:**

1 Jiao Zheng, Ph.D.

Associate Professor

Modern Research Center for Traditional Chinese Medicine

School of Chinese Materia Medica

Beijing University of Chinese Medicine, Beijing 100029, P. R. China

Tel/Fax: (86)-10-64286180

E-mail: zj98v2@163.com

2 Pengfei Tu, Ph.D.

Professor and Director

Modern Research Center for Traditional Chinese Medicine

School of Chinese Materia Medica

Beijing University of Chinese Medicine, Beijing 100029, P. R. China

State Key Laboratory of Natural and Biomimetic Drugs

School of Pharmaceutical Sciences

Peking University, Beijing 100191, P. R. China

Tel/Fax: (86)-10-82802750

E-mail: pengfeitu@163.com

*Co-corresponding Authors


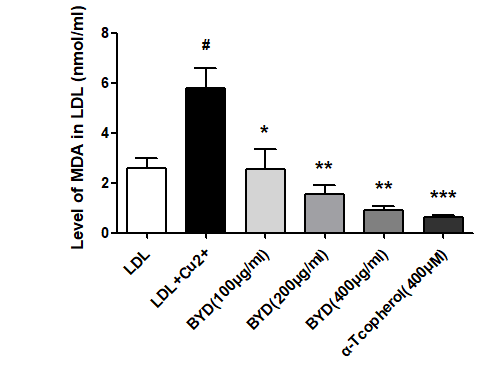


**Figure S1. Inhibition of CuSO4-induced LDL oxidation by BYD.**

The native LDL was pretreated with ultrapure water to 200 μg/mL, and aliquots were preincubated with various concentrations of Baoyuan decoction (100 μg/mL、200 μg/mL and 400 μg/mL, respectively) for 1 h before the addition of CuSO_4_ and compared with the natural antioxidant of α-tocopherol. Then, CuSO_4_ with the final concentration of 10 μM was added and incubated at 37 ℃ for another 24 h. The levels of MDA in lipid peroxidation products were determined according to the specification of the kit (Nanjing Jiancheng Bioengineering Institute, Nanjing, China) (n = 4).


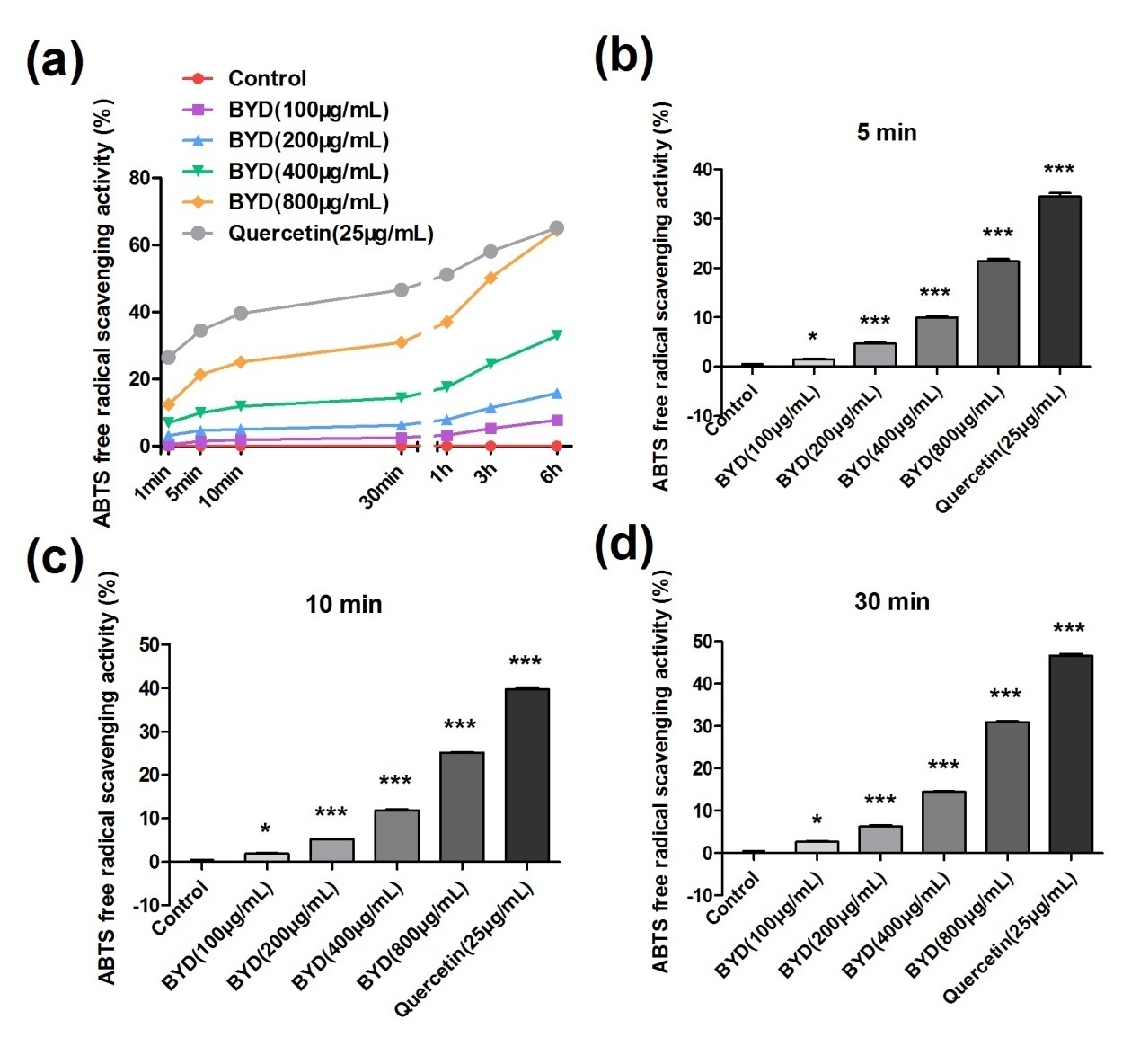


**Figure S2 Scavenging Effect on 2,2'-azino-bis (3-ethylbenzthiazoline-6-sulphonic acid) (ABTS^+^) Radicals.**

**(a)** BYD significantly increased the scavenging of free radical in a dose-dependent manner. ABTS free radical scavenging activity at 5 min **(b)**, 10 min **(c)**, and 30 min **(d).** ABTS^+^ free radical scavenging effect was measured at 734nm by the commercial assay kit (Beyotime Biotechnology, Shanghai, China). Results were expressed by the proportion of ABTS^+^ scavenging activity compared with the control. Quercetin was used as the standard antioxidant (n = 3—4).

**Figure S3 Scavenging Effect on 1,1-Diphenyl-2-Picrylhydrazyl (DPPH) Radicals.**

The DPPH free radical scavenging activity of the extracts was measured according to the method of previously report. 2mL of 0.16mM DPPH radical (Sigma) was added to 2mL of BYD with different concentration (100 to 800 μg/mL); and the quercetin had been used as positive control. After 30min at the room temperature in the dark, then the absorbance of reaction mixture was measured at 517nm using a spectrophotometer with methanol as the blank. Results were expressed by the proportion of DPPH scavenging activity compared with the control. Quercetin was used as the standard antioxidant (n = 4).


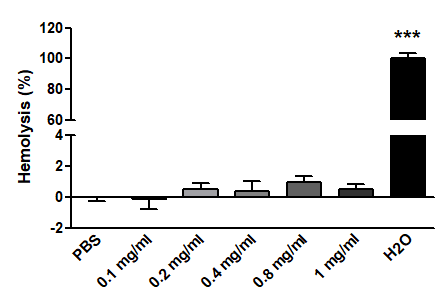


**Figure S4. BYD wasn’t toxic to normal erythrocytes *in Vitro.***

The anti-coagulated blood samples were collected from the healthy C57BL/6 mice. And the stock solution of BYD (20mg/mL) was diluted with PBS to a concentration in the range of 0.1mg/mL to 1mg/mL. Then isobaric erythrocytes were added to each group of them to determine the hemolysis. The complete hemolytic erythrocyte sample, which in distilled water, was used as the positive control, and the nonhemolytic erythrocyte samples under PBS of normal osmotic pressure were used as the negative control (n = 6).
